# Ecology of Floristic Quality Assessment: testing for correlations between coefficients of conservatism, species traits and mycorrhizal responsiveness

**DOI:** 10.1093/aobpla/plx073

**Published:** 2017-12-21

**Authors:** Jonathan T Bauer, Liz Koziol, James D Bever

**Affiliations:** 1 Department of Biology, Indiana University, 1001 E 3rd Street, Bloomington, IN 47405, USA; 2 Kansas Biological Survey, 2101 Constant Avenue, Lawrence, KS 66047, USA; 3 Department of Ecology and Evolutionary Biology, University of Kansas, 1200 Sunnyside Avenue, Lawrence, KS 66045, USA

**Keywords:** Arbuscular mycorrhizal fungi, coefficients of conservatism, disturbance, Floristic Quality Assessment, functional traits, inoculation, succession, tallgrass prairie

## Abstract

Many plant species are limited to habitats relatively unaffected by anthropogenic disturbance, so protecting these undisturbed habitats is essential for plant conservation. Coefficients of conservatism (C values) were developed as indicators of a species’ sensitivity to anthropogenic disturbance, and these values are used in Floristic Quality Assessment as a means of assessing natural areas and ecological restoration. However, assigning of these values is subjective and improved quantitative validation of C values is needed. We tested whether there are consistent differences in life histories between species with high and low C values. To do this, we grew 54 species of tallgrass prairie plants in a greenhouse and measured traits that are associated with trade-offs on the fast-slow continuum of life-history strategies. We also grew plants with and without mycorrhizal fungi as a test of these species’ reliance on this mutualism. We compared these traits and mycorrhizal responsiveness to C values. We found that six of the nine traits we measured were correlated with C values, and together, traits predicted up to 50 % of the variation in C values. Traits including fast growth rates and greater investment in reproduction were associated with lower C values, and slow growth rates, long-lived leaves and high root:shoot ratios were associated with higher C values. Additionally, plants with high C values and a slow life history were more responsive to mutualisms with mycorrhizal fungi. Overall, our results connect C values with life-history trade-offs, indicating that high C value species tend to share a suite of traits associated with a slow life history.

## Teaser

Coefficients of conservatism (C values) are numeric values assigned to plant species to indicate their sensitivity to anthropogenic disturbance, and these values are increasingly used to prioritize natural areas for conservation and monitor restoration outcomes. However, assigning these values is subjective, and quantitative links between C values and a plant species' ecology are needed. We grew seedlings of 54 plant species in a greenhouse and measured traits that are related to life history trade-offs. We found that these traits were correlated with C values, indicating that coefficients of conservatism are closely linked to a plant species' life history strategy.

## Introduction

Floristic Quality Assessment (FQA) is increasingly used to identify natural areas for conservation, monitor outcomes of ecological restoration and evaluate environmental mitigation efforts. Floristic Quality Assessment is based on coefficients of conservatism (C values). These values, between 0 and 10, are assigned to each species of a region’s flora by expert botanists to indicate a species’ reliance on communities that are relatively free of anthropogenic disturbance. With these values, metrics of floristic quality can be calculated from inventories of a site’s flora (Swink and Wilhelm 1994). Frequently, this calculation incorporates both species richness and the mean C value of the species occurring at a site, though other metrics are often used, including the mean C values of all species occurring at a site or the mean C value weighted by species abundance (Swink and Wilhelm 1994).

Correctly assigning C values is important because of FQA’s role in conservation. Floristic Quality Assessment was originally developed as a means to prioritize natural areas for conservation (Swink and Wilhelm 1994), and FQA has since been used to evaluate the outcomes of ecological restoration (Taft *et al.* 2006; Matthews *et al.* 2009, 2014; Spyreas *et al.* 2012). Floristic Quality Assessment is also used in assessments of wetland mitigation in the Chicago region, where FQA was first developed (U.S. Army Corps of Engineers, Chicago District 2009), and several US EPA-supported studies have developed this approach for monitoring of wetland mitigation projects in other regions (e.g. Milburn *et al.* 2007; Rocchio 2007; Bried *et al.* 2012). The widespread acceptance and application of FQA and C values suggests that these metrics reflect the goals of conservation practitioners, but we have a limited understanding of the ecology underlying the assignment of these values.

Following the increasing application of FQA, there have been several attempts to empirically validate these metrics. Matthews *et al.* (2014) found that species with high C values tended to co-occur with other species with high C values, suggesting ecological similarities among species with similar C values. Additionally, Matthews *et al.* (2009) found that the Floristic Quality Index and mean C tend to increase over time following restoration. Correspondingly, Bried *et al.* (2013) found that mean C declines with increasing anthropogenic disturbance. However, there remains a need to better link C values with the underlying biology of the plant species that these values are assigned to.

C values are assigned almost solely based on expert opinion of regional botanists, and the specific criteria used to assign these values vary among authors. Swink and Wilhelm (1994) were the first to include C values and FQA for their flora, and they indicate that C values reflect a species fidelity to communities that are undisturbed by anthropogenic activities. Sensitivity to anthropogenic disturbance is typically the primary consideration in assigning C values (Swink and Wilhelm 1994; Taft *et al.* 1997; Bernthal and Cochrane 2003; Rothrock and Homoya 2005) but other considerations are made by some authors, including habitat specialization or rarity within the region of concern (Bernthal and Cochrane 2003; Rothrock and Homoya 2005). Since C values are intended to indicate a species’ tolerance of anthropogenic disturbance, it is likely that these values reflect a plant species’ life history. Taft *et al.* (1997) made the most explicit link between the values assigned and a plant species’ ecology, with low C values expected for ruderal species and high C values expected for late-successional competitive or stress-tolerant species (Taft *et al.* 1997). The relationship between C values and life history has been tested with limited data sets (Bauer *et al.* 2015), but, to date, the association between C values and life-history strategies has yet to be comprehensively assessed.

In our study, we test for an association between species traits and their coefficients of conservatism. It has long been hypothesized that traits are strongly linked to a plant’s life history (Pianka 1970), and correlations among traits have been linked to trade-offs between a fast and slow life-history strategy (Reich 2014; Salguero-Gómez *et al.* 2016). A fast life history is associated with rapid growth rates, short lifespans and fitness closely tied to reproduction, while a slow life history is associated with slow growth rates, a long lifespan and fitness reliant on survival of established plants (Salguero-Gómez *et al.* 2016). The usefulness of traits as indicators of life histories has been validated by comparisons of species traits with species’ mortality rates and matrix population models (Poorter *et al.* 2008; Adler *et al.* 2014; Salguero-Gómez *et al.* 2016). While consistent across many species, these relationships are not strong enough that a single trait might be used as an indicator of a plant’s life history. However, the potential trait combinations expressed by plant species represent a relatively narrow range of all possibilities (Martínez-Vilalta *et al.* 2010; Reich 2014; Díaz *et al.* 2016), which suggests that several important traits are associated with broad patterns of life-history trade-offs. Thus, by considering multiple traits, we might better characterize a species’ overall life-history strategy.

Several of the traits we examine (e.g. relative growth rate, investment in reproduction) are well-known correlates of life-history strategy. Additionally, in our study system, interactions with arbuscular mycorrhizal fungi (AMF) have been shown to vary with a species’ life history, with late-successional plant species showing greater growth rates in soils inoculated with AMF than in sterile controls (Koziol and Bever 2015). Reliance on the AMF mutualism could become a liability in areas where anthropogenic soil disturbance and eutrophication have degraded soil microbial communities (Johnson *et al.* 1997; Helgason *et al.* 1998), so it is possible that the benefits a plant receives from mutualisms with AMF will be correlated with C values.

Our overall goal was to test for a link between coefficients of conservatism (C values) and a plant species life-history strategy. Our specific objectives were to (i) characterize plant species life histories using functional traits, (ii) test the relationship between these functional traits and C values, (iii) test if AMF responsiveness is associated with C values or plant functional traits and (iv) determine the traits that are most important to determining C values. Lastly, we test whether C values from our study region (Indiana) are correlated with C values from neighbouring regions to determine if our results are likely to be generalizable beyond our study region.

## Methods

To measure plant traits, we grew 54 species of tallgrass prairie plants under common conditions in a greenhouse. We chose species based on availability from local seed nurseries and shared habitat preferences (i.e. mesic tallgrass prairie). We also chose species that thoroughly represented the full range of C values (0–10). These included primarily native species but also ruderal, non-native species that have become important early-successional species. We purchased Indiana genotype seeds produced in nurseries from wild-collected material by Spence Restoration Nursery (Muncie, Indiana). To better represent low C value species in our analysis, we also collected seeds from wild populations of eight species that were unavailable commercially. Seeds were cold-moist stratified for 30 days in sterilized sand, then transferred to sterilized potting soil to germinate. After true leaves had formed on seedlings of all species, seedlings were transplanted into 1 L ‘D60 Deepots’ (Stuewe & Sons Inc.) that were filled with pasteurized topsoil (Crider silt-loam) mixed 50:50 with coarse river sand to facilitate drainage and the recovery of plant roots. The pots were first filled with 800 mL of soil. Then, half of the pots were inoculated with 50 mL of AMF inoculum containing a diversity of species previously demonstrated to increase plant growth (Bauer *et al.* 2012; Koziol *et al.* 2015; Middleton *et al.* 2015). While we know that individual plant species vary in their response to AMF species (Koziol and Bever 2016a), we generally find that response to a mixture of AMF species is similar to the effect of the fungi that is most effective at promoting plant growth (Vogelsang *et al.* 2006; Koziol and Bever 2016b). All pots were then capped with a layer of pasteurized soil. Each species was grown in five replicates of inoculated and control soils with 10 seedlings total per species, and seedlings were allowed to grow in the greenhouse for 16 weeks before the full plant was harvested and traits were measured.

We measured seven traits at harvest for each individual in our experiments, and we measured two additional traits on seeds for each of our study species. Measurements were made on seeds from the nursery, or from wild-collected seeds for those species that were unavailable commercially. Each trait was selected because of hypothesized relationships with a plant species’ life history (Table 1). We measured total biomass at the conclusion of the experiment, as an indication of relative growth rates. Harvested plants were separated into flowering biomass, other above-ground biomass and roots to calculate root:shoot ratios and flowering biomass as a percentage of total biomass. At harvest, we also recorded the total number of leaves produced (including dead leaves), and plant height as indicators of how plant growth was allocated. To characterize leaves, we measured the thickness of the leaves, and we recorded the ratio of dead leaves to the total number of leaves produced by the plant. To characterize seeds, we weighed at least 20 seeds per species to determine average seed mass, and we measured deviation from a spherical shape, calculated as the variance in measures of the length, width and height of the seed. We measured mycorrhizal responsiveness as the total biomass of seedlings in inoculated soils divided by the biomass of seedlings grown in control soils as an indicator of the benefits a plant species derive from this mutualism. Methods primarily follow (Pérez-Harguindeguy *et al.* 2013) with detailed rationale in Table 1. We note that 16 weeks is less than a full growing season, so our results reflect the traits of young plants and the life-history trade-offs involved in early growth and establishment. It is likely that the relationships we describe here would be different if traits were measured over a longer experiment or on mature plants under field conditions.

**Table 1.** Hypothesized links between traits and plant species’ life history and our predictions for relationships between these traits and C values. Overall, the traits we measured can be good indicators of a plant species life history and successional association. We predict that species with an early-successional life history will tend to have low C values, and species with a late-successional life history will tend to have high C values.

Trait	Prediction	References
Total biomass	Rapid growth is associated with a ruderal life history, so we expect slower growth rates among species with high C values.	Hunt and Cornelissen (1997)
Root:shoot	The fitness of late-successional plants is more dependent on the survival of established plants, and investment below-ground is likely to be necessary to maintain the high competitive ability of late-successional plants. Consequently, we predicted higher root:shoot ratios among species with high C values.	Monk (1996); Tilman and Wedin (1991); Craine *et al.* (2003)
% Flowering biomass	Early-successional plants are expected to invest more resources in reproduction and to allocate resources to reproduction earlier than late-successional plant species. We predicted more investment in reproductive biomass among species with low C values.	Pianka (1970)
Number of leaves	We expected that fast-growing ruderal species would produce more leaves due to fast growth rates and to compete for light with other fast-growing ruderal species. We expected more leaves among species with low C values.	
Proportion of dead leaves	This measurement was taken as an indicator of leaf lifespan, and, together with leaf thickness, these data were collected as an indication of the ‘leaf economic spectrum’. Early-successional plants are expected to invest relatively few resources in thin leaves, maximizing photosynthetic area to capture high amounts of light, but these leaves are short-lived. In contrast, late-successional plants are expected to invest more resources in long-lived leaves.	Wright *et al.* (2004); Adler *et al.* (2014)
Leaf thickness	Predictions for this trait follow proportion of dead leaves. We expected species with high C values to have both thicker leaves and a lower proportion of dead leaves.	Wright *et al.* (2004); Adler *et al.* (2014)
Height	Adult height can be associated with late-successional plant species’ competition for light. However, this may not be the case in grasslands, especially among seedlings. We expected that early-successional species would grow taller as an adaptation to competing for light alongside other fast-growing species in disturbed environments.	Tilman (1985); Reynolds and Pacala (1993)
Variation in seed dimensions	Low variation in seed dimensions indicates a spherical seed, and this is expected to facilitate the burial of seeds in the seed bank. This may indicate a species reliance on the seed bank for future recruitment, a strategy that we hypothesized would be important to early-successional species. We predicted that species with low C values may have more spherical seeds.	Thompson *et al.* (1993); Bekker *et al.* (1998)
Seed mass	Especially in forests, high seed mass may be required to recruit into highly competitive environments, so we predicted greater seed mass among late-successional species.	Adler *et al.* (2014)

### Data analysis

To identify the most important axes of variation in plant traits, we conducted a principal component analysis (PCA) on the average trait values (averaged across AMF treatments) for each of our study species. We expected larger differences in traits between early- and mid-successional than between mid- and late-successional plant species, so we first ln-transformed trait values to linearize the relationship between traits and successional associations (Koziol and Bever 2016a). Since traits were measured in different units, we standardized all values on a scale from 0 to 1 by subtracting the minimum value for all species from the mean value for each species and dividing by the range of values (the maximum value for each trait minus the minimum). Using this data we conducted a PCA using prcomp in R.

We conducted a multiple regression to identify the principal components (PCs) that were correlated with species’ C values. For our analyses, we use the C values assigned to the flora of Indiana (Rothrock and Homoya 2005), since the seeds used in our experiments all originated from Indiana. After identifying the first axis as predicting the most variation in trait data and as the best correlated with C values, we used linear regression to test the association between the first PC of trait values and mycorrhizal responsiveness. Mycorrhizal responsiveness was calculated by dividing the average biomass of individuals grown in inoculated soils by the biomass of individuals grown in pasteurized, non-inoculated soil. Mycorrhizal responsiveness data were ln-transformed to meet assumptions of statistical tests and so that positive values indicate species that benefit from AMF and negative values indicate species with higher growth rates in pasteurized soil.

To further identify the relative importance of each of our nine measured traits and AMF responsiveness as predictors of species’ C values, we analysed the correlations between each trait and C values separately. Then, we used model averaging to identify the traits that are the most important predictors of C values using the model.ave function in the MuMIn package in R. Initially, all possible linear additive models were tested using the dredge function in MuMIn, including or excluding all nine measured traits and mycorrhizal responsiveness. For model averaging, we retained all models with ΔAIC < 4.

To explore the generality of our results, and because of the subjective nature of C values, we tested for correlations between C values assigned to the flora of Indiana and that of neighbouring regions that also contain substantial areas of tallgrass prairie.

## Results

**Figure 1.**
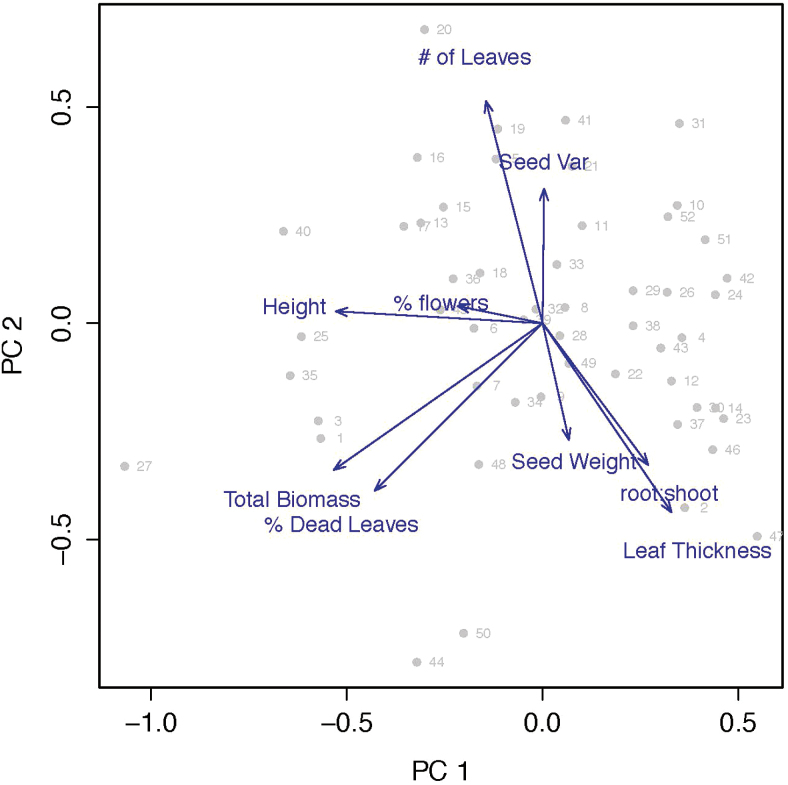
Principal component analysis with points representing species and blue arrows representing trait values. Numbers correspond to species names identified in Table 3. PC1 best represents variation in species life history, with six traits loading strongly on this axis. Short-lived species known to benefit from disturbance are associated with low values on PC1, and long-lived species associated with undisturbed prairies are associated with high values on PC2 (Table 2).

The first axis of the PCA separates species that are fast growing, invest more in reproduction, have short-lived leaves and grow taller from plant species that have high root:shoot ratios and thick leaves. The second axis separates species that have many leaves and irregularly shaped seeds from species that have heavy seeds. These two axes explain 49 % of the variation in measured traits among these species (Fig. 1). PC3 explains an additional 14 % of the variation in traits, with root:shoot and percent flowering biomass loading most strongly onto this axis (Table 2). A full species list, PC scores and C values are included in Table 3.

**Table 2.** Results of our PCA of species traits.

			Trait loadings								
	Proportion of variance	Cumulative proportion of variance	Root:shoot	% Flowering biomass	Total biomass	Leaf thickness	Height	No. of leaves	% Dead leaves	Seed weight	Variation in seed dimensions
PC1	0.30	0.30	0.27	−0.22	−0.53	0.33	−0.53	−0.14	−0.43	0.07	0.00
PC2	0.19	0.49	−0.33	0.04	−0.34	−0.44	0.03	0.51	−0.39	−0.27	0.31
PC3	0.14	0.63	0.65	−0.44	0.43	−0.24	−0.07	0.20	−0.11	−0.24	0.20
PC4	0.11	0.73	−0.27	−0.05	0.45	0.39	0.17	−0.05	−0.55	0.28	0.40
PC5	0.09	0.82	0.20	0.15	−0.22	−0.29	0.03	−0.33	0.24	0.37	0.71
PC6	0.07	0.89	−0.30	−0.01	0.18	−0.08	−0.30	−0.57	0.00	−0.64	0.19
PC7	0.06	0.94	0.00	0.08	0.15	−0.62	−0.09	−0.34	−0.44	0.34	−0.40
PC8	0.03	0.98	−0.33	−0.85	−0.18	−0.10	0.24	−0.15	0.15	0.15	−0.03
PC9	0.02	1.00	−0.31	−0.09	0.28	−0.08	−0.73	0.31	0.29	0.32	0.06

**Table 3.** Species number as labelled in Fig. 1, list of species included in our experiment, their position on our first two PCA axes and their C value in Rothrock and Homoya (2005).

Number	Species name	PC1	PC2	CC
1	*Abutilon theophrasti*	−0.57	−0.27	0
2	*Allium cernuum*	0.36	−0.43	4
3	*Ambrosia artemisiifolia*	−0.57	−0.23	1
4	*Amorpha canescens*	0.36	−0.03	9
5	*Andropogon virginicus*	−0.12	0.38	1
6	*Asclepias incarnata*	−0.18	−0.01	4
7	*Asclepias syriaca*	−0.17	−0.15	1
8	*Asclepias tuberosa*	0.06	0.04	4
9	*Asclepias verticillata*	0.00	−0.17	5
10	*Aster laevis*	0.34	0.27	10
11	*Aster novae-angliae*	0.10	0.23	3
12	*Baptisia alba*	0.33	−0.13	10
13	*Bidens bipinnata*	−0.31	0.23	2
14	*Cacalia atriplicifolia*	0.44	−0.20	6
15	*Carex scoparia*	−0.25	0.27	4
16	*Carex tribuloides*	−0.32	0.38	5
17	*Carex vulpinoidea*	−0.35	0.22	2
18	*Chamaecrista fasciculata*	−0.16	0.12	2
19	*Chenopodium album*	−0.11	0.45	0
20	*Conyza canadensis*	−0.30	0.68	0
21	*Coreopsis tripteris*	0.08	0.36	8
22	*Desmodium illinoense*	0.19	−0.12	5
23	*Echinacea pallida*	0.46	−0.22	10
24	*Echinacea purpurea*	0.44	0.07	6
25	*Elymus canadensis*	−0.62	−0.03	5
26	*Eryngium yuccifolium*	0.32	0.07	10
27	*Setaria faberi*	−1.07	−0.33	0
28	*Helianthus grossesseratus*	0.04	−0.03	3
29	*Heliopsis helianthoides*	0.23	0.08	4
30	*Helianthus occidentalis*	0.39	−0.19	9
31	*Koeleria cristata*	0.35	0.46	8
32	*Lespedeza capitata*	−0.02	0.03	4
33	*Monarda fistulosa*	0.04	0.14	3
34	*Oenothera biennis*	−0.07	−0.18	0
35	*Panicum capillare*	−0.64	−0.12	0
36	*Panicum virgatum*	−0.23	0.10	4
37	*Parthenium integrifolium*	0.34	−0.23	9
38	*Penstemon digitalis*	0.23	−0.01	4
39	*Physostegia virginiana*	−0.05	0.01	5
40	*Polygonum pennsylvanicum*	−0.66	0.21	0
41	*Pycnanthemum virginianum*	0.06	0.47	5
42	*Ratibida pinnata*	0.47	0.10	5
43	*Rudbeckia hirta*	0.30	−0.06	2
44	*Rumex crispus*	−0.32	−0.78	0
45	*Schizachyrium scoparium*	−0.26	0.03	4
46	*Silphium integrifolium*	0.43	−0.29	7
47	*Silphium terebinthinaceum*	0.55	−0.49	6
48	*Sorghastrum nutans*	−0.16	−0.33	4
49	*Sporobolus heterolepis*	0.07	−0.09	10
50	*Tradescantia ohiensis*	−0.20	−0.72	3
51	*Vernonia altissima*	0.42	0.19	2
52	*Veronicastrum virginicum*	0.32	0.25	8

**Figure 2.**
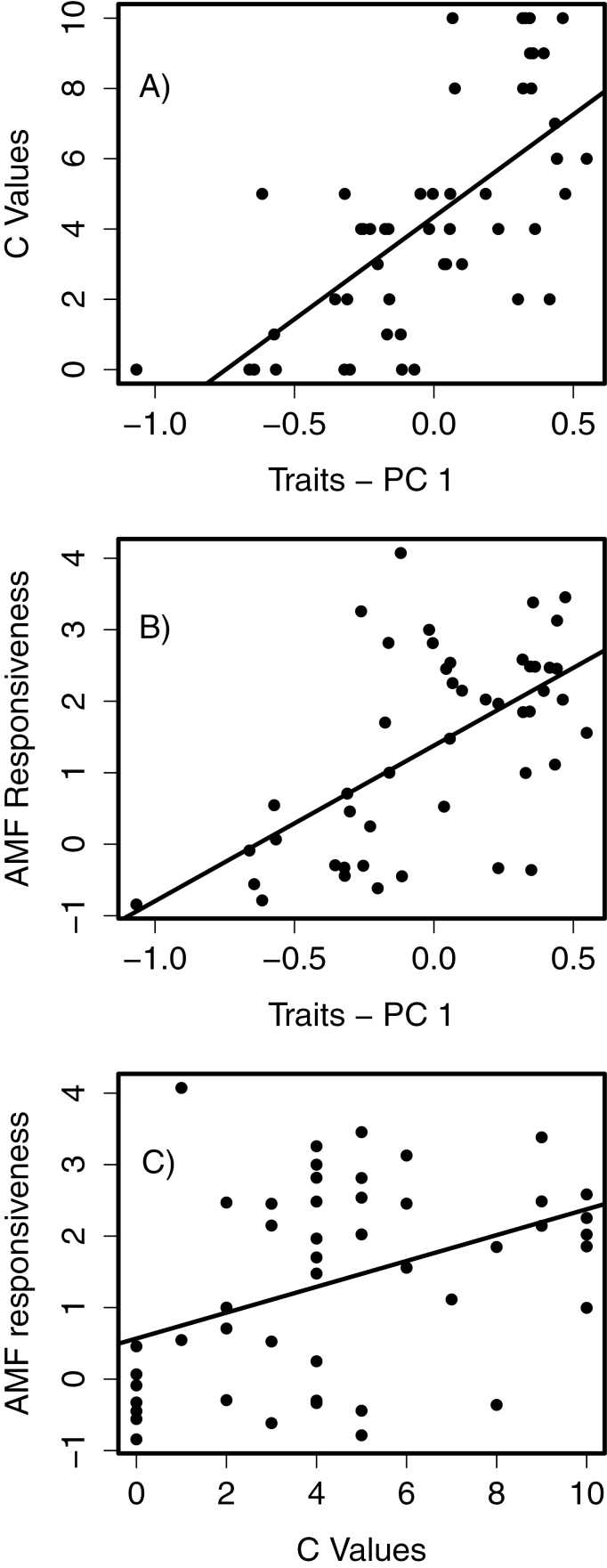
(A) C values and the first PC of species traits (Fig. 1) are significantly correlated (DF = 50, *y* = 5.8*x* + 4.3, *P* < 0.001, *r*^2^ = 0.45). Low scores on PC1 are correlated with traits typically of an early-successional life history and high scores are associated with late-successional traits. C values are from Rothrock and Homoya (2005). (B) Responsiveness of plant species to AMF as compared to the first PC of species traits (DF = 45, *y* = 1.38*x* + 1.38, *P* < 0.001, *r*^2^ = 0.35). (C) Species with high C values tend to be more responsive to mycorrhizal fungi (DF = 45, *y* = 0.18*x* + 0.57, *P* = 0.004, *r*^2^ = 0.17).

We tested all PCs as predictors of C values and identify the first PC as statistically significant (*P* < 0.001), while the remaining PCs were not significant predictors of C values. PC1 is positively correlated with C values (*r*^2^ = 0.45; Fig. 2A) and with a plant species’ responsiveness to AMF (*r*^2^ = 0.35; Fig. 2B), and AMF responsiveness is positively correlated with C values (*r*^2^ = 0.17; Fig. 2C).

**Figure 3.**
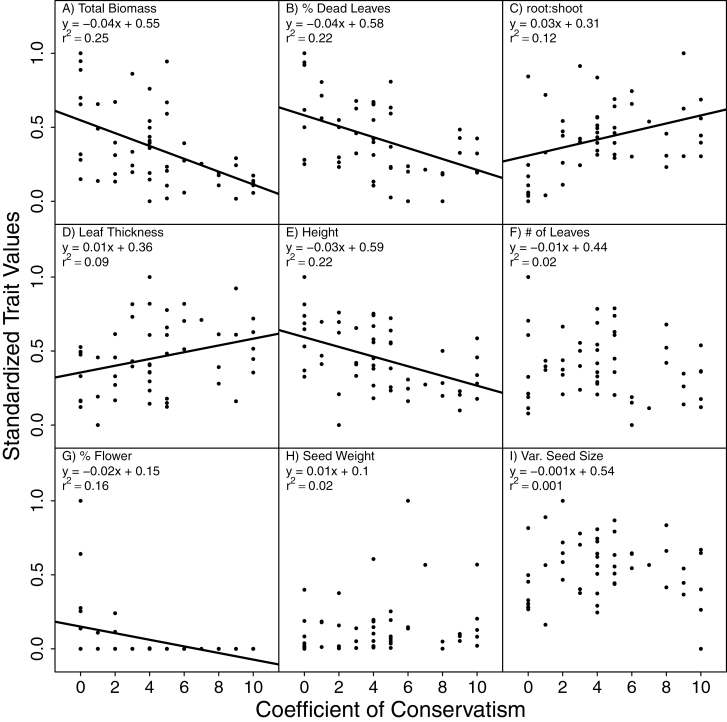
Plots of individual traits as compared to coefficients of conservatism. Traits were log-transformed and then standardized on a scale of 0–1, as in the PCA. We hypothesized that total biomass, percentage of dead leaves, height, number of leaves and percentage of biomass invested in reproduction (A, B, E, F and G) are indicators of an early-successional life history and that these traits would be negatively correlated with C values. In contrast, we hypothesized that root:shoot, leaf thickness and seed weight (C, D and H) would indicate a late-successional life history and be positively correlated with C values. Regression lines are displayed for significant relationships (DF = 50, *P* < 0.05).

**Figure 4.**
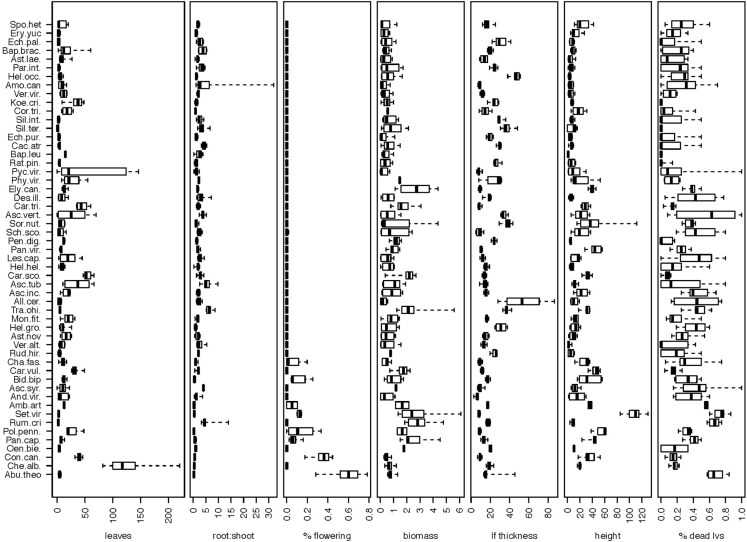
Intraspecific variation in species traits with central dark lines representing medians, boxes representing interquartile range and whiskers representing minimum and maximum values. Species are arranged from highest C values at top to lowest C values at the bottom of the figure. Data include traits measured on plants grown with and without mycorrhizal fungi, so variation is increased in traits that are responsive to mycorrhizal inoculation (including total biomass) since the data here include both within and between treatment variation.

When analysed independently, six of the nine traits we measured were significant predictors of C values (Fig. 3, with intraspecific variation in traits displayed in Fig. 4). These results are consistent with the PCA, which identified these six variables as loading strongly on the first PC (Table 2). In tests of the relative importance of these traits and AMF responsiveness as predictors of C values, 28 models were retained with similar fits to the data (ΔAICc < 4). Model averaging indicated that total biomass, percent dead leaves and root:shoot ratio were retained in most models and these variables were identified as statistically significant predictors of C values. Similar to the PCA, these three variables together were good predictors of C values (*r*^2^ = 0.50). However, plant height, proportion of flowering biomass, leaf thickness and AMF responsiveness were not retained in the top models and were not statistically significant predictors of C values after including the three top variables (Table 4). This reduction in total variables is consistent with correlations among plant functional traits that are associated with a plant species’ life-history strategy (Reich 2014).

**Table 4.** Model averaging results for the nine traits we measured in our experiment plus mycorrhizal responsiveness as predictors of C values.

	Importance	N containing models	Estimate	SE	*P*
Intercept			6.9	1.9	0.0003
Total biomass	0.98	27	−4.1	1.6	0.0108
Percent dead leaves	0.98	27	−4.1	1.6	0.0137
Root:shoot	0.92	25	4.2	1.7	0.0167
No. of leaves	0.66	16	−3.1	1.6	0.0624
Percent flowering biomass	0.49	14	−3.9	2.5	0.1173
Leaf thickness	0.20	6	1.7	1.5	0.2761
Variation in seed dimensions	0.18	7	−1.6	1.7	0.3705
AMF responsiveness	0.12	5	−0.1	0.6	0.8058
Seed weight	0.10	4	1.1	1.9	0.5659
Height	0.08	3	1.0	2.5	0.7015

We found strong correlations between C values assigned by different authors (*r*^2^ = 0.68–0.81; Table 5), suggesting general agreement among authors about appropriate C values.

**Table 5.** *r* ^2^ values for correlations among C values assigned to neighbouring regions, including Indiana (Rothrock and Homoya 2005), Chicago (Swink and Wilhelm 1994), Illinois (Taft 1997), Wisconsin (Bernthal and Cochrane 2003) and Michigan (Herman *et al.* 1997). The Chicago region includes counties in Illinois, Wisconsin, Indiana and Michigan, but we include the C values from the Chicago region because different authors assigned values in each region.

	Chicago	Illinois	Wisconsin	Michigan
Indiana	0.76	0.77	0.69	0.68
Chicago		0.86	0.68	0.67
Illinois			0.81	0.68
Wisconsin				0.74

## Discussion

Our findings provide insight into the ecology underlying coefficients of conservatism (C values) and FQA. Despite their widespread use, C values have been criticized as subjective. One response to this concern has validated C values by showing the consistent co-occurrence of conservative plant species (Matthews *et al.* 2014), and others have shown declines in average C values of plant species in communities impacted by anthropogenic disturbance (Cohen *et al.* 2004; Bried *et al.* 2013). This work suggests shared ecological characteristics of conservative plant species. We confirm this possibility by identifying correlations among plant species’ traits, mycorrhizal responsiveness and C values. We chose traits that are associated with a species’ life-history strategy, so the patterns we observe strongly link C values and FQA with the study of life-history trade-offs.

**Figure 5.**
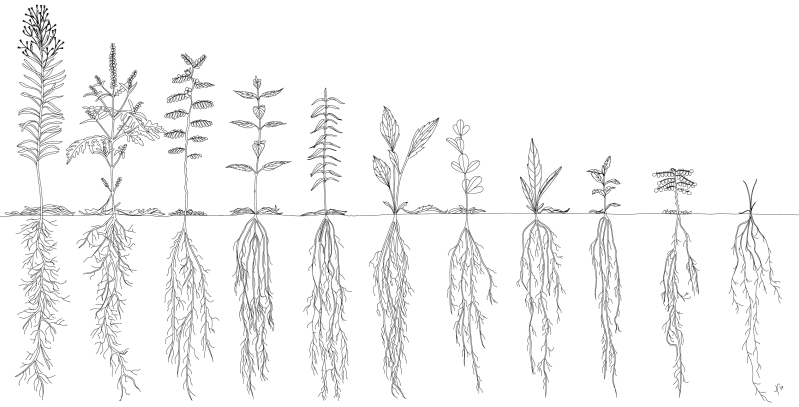
Illustration of representative plant species across a range of coefficients of conservatism. Plant height, percent dead leaves, root biomass, root:shoot and percent flowering biomass are all scaled based on regressions of the untransformed data. Rooting width and depth was constrained by pot size. Since all species grew through the full depth of the pot by the end of the experiment, roots are illustrated as being the same length. Species illustrated include (from left to right): *Conyza canadensis*, *Ambrosia artemisiifolia*, *Chamaecrista fasciculata*, *Monarda fistulosa*, *Asclepias tuberosa*, *Ratibida pinnata*, *Baptisia alba*, *Silphium integrifolium*, *Veronicastrum virginicum*, *Amorpha canescens* and *Sporobolus heterolepis*.

In our PCA, the first axis appears to represent the fast-slow continuum of life-history variation (Reich 2014; Salguero-Gómez *et al.* 2016). Traits associated with a fast life history, including rapid growth rate and early investment in reproduction, tend to be associated with low values on PC1. Traits associated with a slow life history, including high root:shoot ratio and long-lived leaves, are associated with higher values on PC1 (Fig. 5). Correspondingly, species with low values on PC1 are generally short-lived species that are typically found in disturbed habitat (e.g. *Setaria faberi* and *Ambrosia artemisiifolia*), and long-lived species associated with relatively undisturbed habitat tend to have higher values on PC1 (e.g. *Silphium terebinthinaceum* and *Echinacea pallida*). The correlation between PC1 and C values provides strong support for our prediction that C values would be associated with a plant species’ life history. Specifically, species with a fast life history tend to be assigned low C values, while plant species with a slow life history tend to be assigned high C values.

These life-history trade-offs can be linked to the shifts in species abundance that occur during succession (Connell and Slatyer 1977). Successional associations are not always explicitly considered when assigning C values (but see Taft 1997). Instead, C values are intended to reflect a species’ reliance on communities that are relatively free of anthropogenic disturbance (Swink and Wilhelm 1994). However, it is likely that the successional status of species associations was implicitly considered while assigning C values, since communities experiencing high levels of anthropogenic disturbance are likely to be dominated by early-successional species. Our results are consistent with Taft’s arguments that C values reflect species’ successional associations (Taft 1997), with low C value species being early successional and high C value species being late successional.

Although our analysis finds a strong relationship between C values and species traits, there is substantial residual variation to explain. C values are meant to indicate a plant species tolerance of anthropogenic impacts to natural areas, and there are some cases where this might lead C values to diverge from the predictions we would make from species traits. For example, some late-successional species could be resistant to anthropogenic disturbance and, therefore, be assigned a relatively low C value. In contrast, some ruderal species could have high C values because they rely on natural disturbances that have been altered by human activities. Additionally, there is some variation among authors in values assigned to some species that may reflect deviations from a species’ true sensitivity to anthropogenic disturbance and contribute to the residual variation in our analyses (Table 4). Furthermore, the traits we measured are an imperfect reflection of the mechanisms underlying life-history trade-offs, since these traits are primarily ‘soft traits’ rather than traits like competitive ability, plant–soil feedbacks, dispersal abilities, etc. that represent the true mechanisms underlying life-history trade-offs. Moreover, we only test for simple life-history trade-offs in linear relationships between C values and plant traits. However, life-history strategies can be multidimensional (Herrera-Peraza *et al.* 2016), and there is likely to be substantial variation in species traits that does not fit neatly into our conceptual or statistical models. Additional studies with larger data sets that explore potential non-linear relationships among life-history traits (e.g. Fig. 3I) and a more nuanced approach to characterizing relationships between life history and C values may generate additional insights into C values and FQA. Nonetheless, given the inherent limitations of our approach, we find it remarkable that our measured traits explain up to 50 % of the variation in C values.

Past work has indicated that early- and late-successional plant species differ in their interactions with soil communities (Bauer *et al.* 2015), and Koziol and Bever (2015) identify a trade-off between relative growth rates and mycorrhizal responsiveness. Our results provide further insight into these patterns by associating mycorrhizal responsiveness with a suite of traits that are linked to a species’ life-history strategy. Plant species that tend to invest less in rapid growth and reproduction tend to invest more in long-lived leaves, higher root:shoot ratios and benefit more from mutualisms with AMF. Overall, species traits are a better predictor of a species’ C value than AMF responsiveness, indicating that species’ sensitivity to anthropogenic disturbance may be better explained by overall life-history strategy regardless of their dependence of AMF.

Nevertheless, the general pattern of dependence of high C value plants on soil symbioses may have important implications for conservation. Anthropogenic impacts to soils, including soil disturbance and fertilization (Johnson *et al.* 1997; Helgason *et al.* 1998), disrupt mycorrhizal communities, so reintroductions of plant species that are strongly responsive to mutualisms with AMF are likely to benefit from re-establishment of this mutualism during restoration efforts (Middleton and Bever 2012; Middleton *et al.* 2015; Koziol and Bever 2016b).

Correlations between C values assigned in our study region and neighbouring study regions suggest that our results are generalizable within the eastern tallgrass prairie, and we expect that our results will be generalizable at a broader geographic scale. However, we also expect cases where ruderal species might be high conservation priorities. Some endangered species that might be considered ruderal species have experienced population declines due to the disruption of natural disturbance regimes. As an example, *Boltonia decurrens* is listed as a threatened species by the USFWS, with population declines linked to the reduction of scouring floods along the Mississippi River (USFWS 1990; Smith *et al.* 2005). In landscapes with long histories of agricultural land use, land abandonment may even produce the opposite pattern that we observe in our study system, where agricultural intensification is relatively recent. For example, in areas of Europe where extensive areas of farmland are being abandoned, some ruderal species associated with agriculture may be among the highest conservation priorities (Gustavsson *et al.* 2007; Moeslund *et al.* 2016). However, in many landscapes, habitat destruction and disturbance continue to increase with intensification of land use (Hoekstra *et al.* 2005), and in these cases we expect the disproportionate loss of late-successional plants.

## Conclusions

We found correlations among plant species’ traits, mycorrhizal responsiveness and the C values assigned to those species. These correlations provide insight into the ecology underlying C values and the metrics derived from these values for FQA. Currently, the assignment of C values is primarily based on expert opinion, and, as a result, is somewhat subjective. Since we measured traits previously shown to be associated with a plant’s life history, our results link C values and FQA with plant life-history trade-offs. We show that traits including relative growth rate, long-lived leaves and root:shoot ratios are correlated with C values, and these traits are known to be reflective of the trade-offs between increasing fitness through greater reproduction vs. greater survival of established individuals (Salguero-Gómez et al. 2016). These trade-offs are referred to as the fast-slow continuum of plant life history, and we show that low C values are associated with a fast life-history strategy and high values are associated with a slow life-history strategy. Further, our results provide additional evidence that interactions with mycorrhizae are an important component of a species’ life history, with slow life history, high C value plants tending to be more responsive to this symbiosis.

## Sources of Funding

This project was supported by the Agriculture and Food Research Initiative Competitive Grant Nos. 2016-67011-25166 (L.K.) and 2016-67012-24680 (J.T.B.) from the USDA National Institute of Food and Agriculture. We also acknowledge support from National Science Foundation DEB 0919434 and 1556664 and from SERDP (RC-2330).

## Contributions by the Authors

J.T.B., L.K. and J.D.B. designed the experiment. J.T.B. and L.K. initiated the experiment, and J.T.B. harvested the greenhouse experiment and collected and analysed data. J.T.B. drafted the manuscript with feedback on revisions from L.K. and J.D.B.

## Conflict of Interest

L.K. is the owner of MycoBloom LLC.
